# Incidence and Risk Factors of Oral Mucositis in Patients with Breast Cancer Who Receiving Chemotherapy in Al-Bashir Hospital

**Published:** 2016-10-01

**Authors:** Ahmed A Al Ibraheemi, Shaimaa Shamoun

**Affiliations:** 1 Clinical Oncology Resident Doctor, Clinical Oncology and Radiation Therapy Department, Al-Bashir Hospital, Al-Ashrafiyah, Amman, Jordan; 2 Oncology Nurse Specialist, Clinical Oncology and Radiation Therapy Department, Al-Bashir Hospital, Al-Ashrafiyah, Amman, Jordan

**Keywords:** Risk factors, Oral mucositis, Breast cancer, Taxane

## Abstract

**Background:** Oral Mucositis (OM) remains the most common side effect of chemotherapy affects negatively on patients' quality of life.

**Subjects and Methods** **:** Convenience samples of patients who received chemotherapy were followed from first or second cycle of chemotherapy until OM occurrence. We reviewed 75 female patients with breast cancer who received chemotherapy with mean age (47.2 SD ± 8.62861). We used WHO scale to assess the severity of OM. Demographic and other variables (age, number of cycle before appearance of signs of OM, WBC count, neutropenia count, creatinine and BMI) were filled in questionnaire.

**Results:** 81.3% of reviewed patients were suffering from OM and (52.4%) of them were shown score 2 according to WHO classification, Taxane included chemotherapy protocol was the only significant variable that associated with OM occurrence (p=0.009).

**Conclusion:** In this study; Taxane is the only risk factor that significantly associated with occurrence of OM.

## Introduction

 Oral Mucositis is a common important sequel of cancer therapy and despite the use of variety of treatment, it is still a major source of additional illness and suffering.^1^^,^^2^ The European Society for Medical Oncology (2007) defined OM as an inflammatory process of oral cavity caused by high dose cancer therapies. Clinically, Oral Mucositis is characterized by generalized erythema, pseudo-membraneous degeneration, frank ulceration and hemorrhage. It is usually observed within 3-5 days after the initiation of chemotherapy and reached peak intensity at 7-14 days, which affects negatively on quality of life (QOL).^3^ By conducting this study; we tried to have better understanding about the relationship between different factors and Oral Mucositis. We also tried to address the role of health team to provide comfortable measures to decrease the occurrence of Oral Mucositis by continuing assessment of oral cavity and utilizing a symptom-oriented approach. Oral Mucositis is the common problem of cancer therapy; most studies have found correlation between Oral Mucositis and quality of life (QOL). It has been shown that OM has devastating impact on patients 'QOL affecting multiple spheres of daily and psychosocial functional; in particular, the functional sphere of QOL was mostly compromised.^4^ Oral Mucositis disrupts the function and integrity of oral cavity, decreases QOL and increases morbidity with pain lead to anorexia, dehydration and malnutrition. Severe OM compromises functional activity such as eating, swallowing, affected social interaction and emotional well-being.^4^ It also increases risk for developing systemic infections, which caused prolong hospitalization and increased economic burden.^5^ This study is considered as the first study to address the incidence of OM with breast cancer in female patients who received chemotherapy in our onco-radiotherapy department in Al-bashir hospital. Beside, this study is contributed to better understanding the relationship between factors (patient-related and cytotoxic therapy-related) that associated with OM occurrence among Jordanian adult breast cancer patients who receiving chemotherapy. This study is designed to achieve the following purposes:

1) Determination the incidence of OM as a side effect of cancer therapy among Jordanian adult breast cancer patients who receive chemotherapy.

2) Description of OM occurrence severity according to WHO scale which exhibited grading of OM.

3) Determination of predisposing factors (patient-related and cytotoxic therapy-related).

## SUBJECTS AND METHODS

 This is a prospective non-experimental cross-sectional designed study. All patients who were newly diagnosed with breast cancer during a period of 4 months (Feb–June, 2015) and who had chemotherapy (Adriamycine, Cychlophosphamid and Taxane) in onco-radiotherapy department in Al-Bashir hospital were included. Non-probability convenience sample of patients who met the inclusion criteria were obtained.

**Inclusion criteria**

-Able to communicate either verbally or in writing and understand (in either Arabic or English).

-18 years of age or older (adult patients).

-Patients with breast cancer who receiving of chemotherapy (Adriamycine, Cychlophosphamid and Taxane) were followed from first or second cycle until Oral Mucositis occurrence.

**Exclusion criteria**

-Those who are mentally incapacitated and those who received radiotherapy or other cancer treatment rather than chemotherapy.

Data was collected from patients admitted in Onco-radiotherapy department in Al-Bashir hospital both in-patients and out-patients with breast cancer and also after the completion of first or second cycle of chemotherapy treatment. Time frame of data collection was taken 4 months (Feb–June) to obtain sufficient sample size. Data was collected by the second author, directly. WHO Oral Mucositis Grading Scale had been adopted to classify the degree of OM and determine these verities of Oral Mucositis. This instrument is widely used in clinical settings and in research studies.^6^ The scale utilizes a 5-grade classification of OM severity.

The scale reads as follows:

Grade 0: no changes (to oral mucosa); Grade 1: soreness, erythema; Grade 2: soreness, erythema, ulceration and ability of eating solid foods; Grade 3: soreness, erythema, ulceration and ability to use a liquid diet only; and Grade 4: soreness, erythema, ulceration and oral alimentation is not possible (Table 1). All independent variables were assessed through a structured interview and through review of patient's record. Demographic and other variables were documented in the questionnaire that was prepared for the study. Those included in (age, number of chemotherapy cycle before appearance of OM signs, WBC count, neutropenia count, creatinine, height and weight and BMI). These data were obtained from the patient's medical record. Patient was asked about his/her smoking status and was measured as number of cigarettes per day. Body Mass Index (BMI) was obtained from the patients records or calculated it by particular equations. The severity of Oral Mucositis was assessed by WHO Oral Mucositis scale.

**Table 1:** WHO Oral Mucositis grading scale

**Grade**	**Description**
**0 (None)**	None
**I (Mild)**	Oral soreness, Erythema
**II (Moderate)**	Oral erythema, Ulcers, Solid diet tolerated
**III (Severe)**	Oral ulcers, liquid diet only
**IV (Life-threatening)**	Oral alimentation impossible

## Results

 A total of 75 patients with breast cancer who received chemotherapy were enrolled in the study. Patients' age was ranged from (31–65 years) with mean of (47.2 SD ± 8.628), patients characteristics were shown in Table 2. We also noticed that there is 56% of patients with age 45 year and less, 60% of patients were receiving (AC) protocol, only 16% of patients were leukopenic and 30.7% of patients were neutropenic. Mean of S. creatinine was (58.87 SD ± 13.19 mmol) and mean of body weight was (76.27 SD ± 16.57 kg).

**Oral Mucositis**

**Figure 1:**
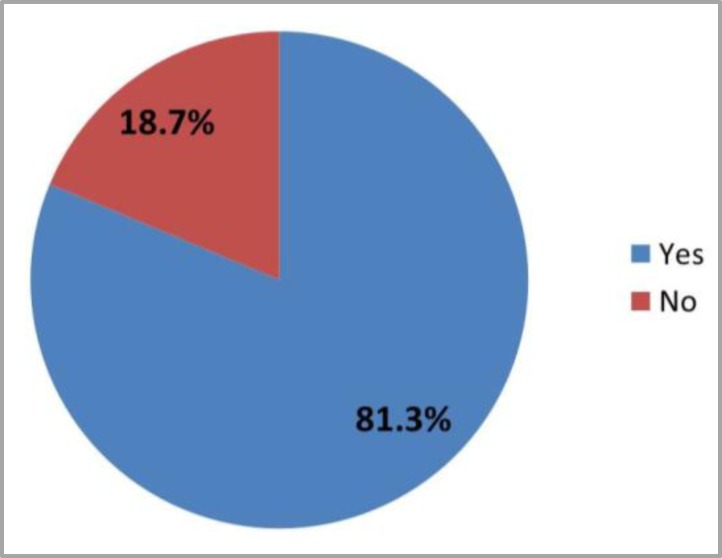
Distribution of patients by presence of OM

**Figure 2:**
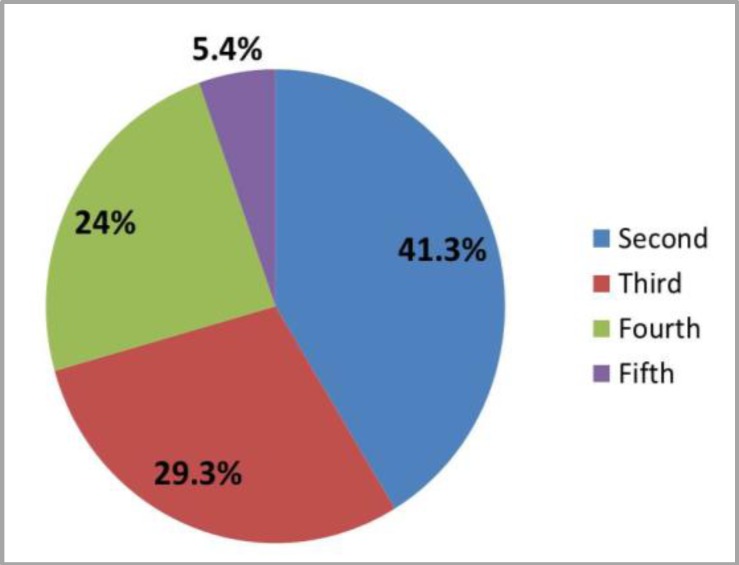
Distribution of patients with OM by cycle of chemotherapy

**Figure 3:**
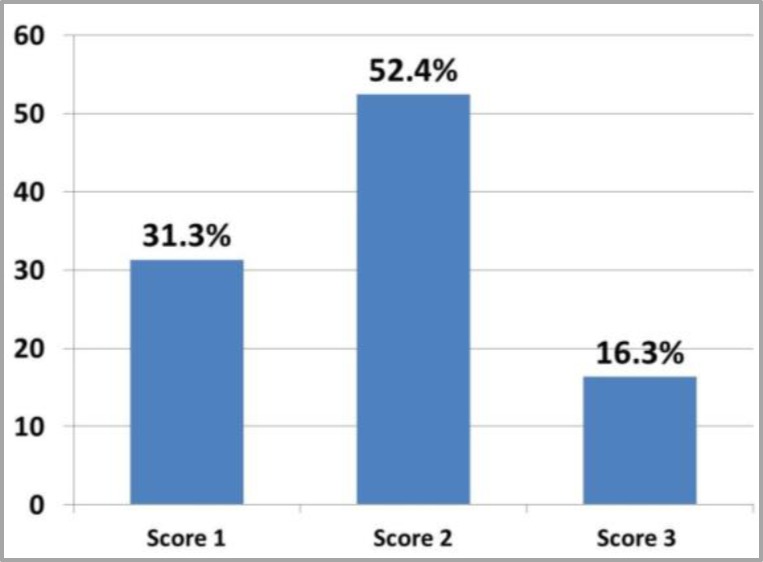
Distribution of OM patients by WHO score of OM

**Figure 4:**
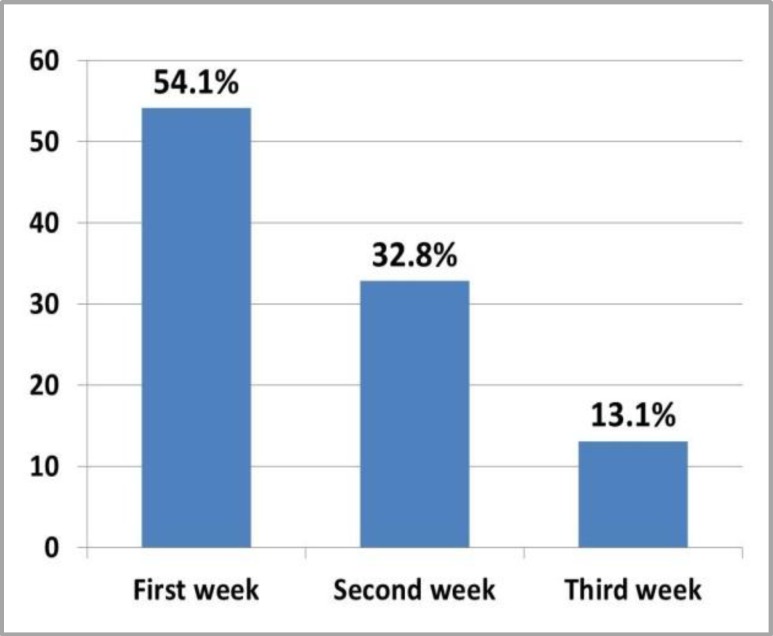
Distribution of OM patients by week of appearance of signs of OM after chemotherapy cycle

Sixty one patients (81.3% ) included in the study were suffering from OM as shown in Figure 1, 41.3% of them were shown OM in the second cycle which is regarded as the highest percentage (Figure 2), more than half (52.4%) of the patients were shown score 2 OM (Figure 3). First week after chemotherapy cycle (which is composed of 3 weeks) was shown the highest percentage of OM appearance which 54.1% of patients suffering from OM (shown in Figure 4).

**Association of OM with different variables**

Table 3 shows association of different variable with OM, in which we noticed that age of the patients was associated with occurrence of OM, 83.3% of patients with age of >45 year were suffering OM but this association was not significant (p=0.41). Taxane included in protocols were associated significantly with occurrence of OM (p=0.009). We declared in Table 3 that 89.3% of patients on Taxane protocols were suffering from OM. Leukopenia and neutropenia were not statistically associated (p=0.6 and 0.12, respectively) with occurrence of OM; whereas 83.3% of leukopenic and 91.3 % of neutropenic patients were suffering from OM.

Approximately, 85% of obese patients included in this study were complained from OM which mean that there is an association between obesity and occurrence of OM but it was not significant (p= 0.6).

There is no significant difference between means of S. creatinine and body weight in OM patients and patients who are not complaining of OM (p=0.892 and 0.532), respectively.

**Table 2:** General characteristics of study sample

**Variable**	**No**	**%**
**Age** **45≥** **45<**	42 33	56 44
**Chemotherapy protocol** **AC** **Taxane**	45 30	60 40
**LeukopeniayesNo**	1263	1684
**Neutropenia** **Yes** **No**	23 52	30.7 69.3
**BMI** **Normal** **Overweight** **Obese**	16 18 41	21.3 24 54.7

**Table 3:** Distribution of patients by occurrence of OM and different variables associated with it

**Variables**	**OM**		**p-value**
	**Yes**	**NO**	
**Age** ** 45≥** **45<**	78.8% 83.3%	21.1% 16.7%	0.41
**Protocol** **AC** **Taxane**	81.4% 89.3%	18.6% 10.7%	0.009
**LeukopeniaYesNo**	83.3%81%	16.7%19%	0.6
**Neutropenia ** **Yes** ** No**	91.3% 76.9%	8.7% 23.1%	0.12
**BMI ** **Normal** **Overweight** **Obese **	75% 77.8% 85.4%	25% 22.2% 14.6%	0.6

Creatinine level was also was shown no significant relation with occurrence of OM (p=0.9).

**Time of OM appearance**

**Figure 5:**
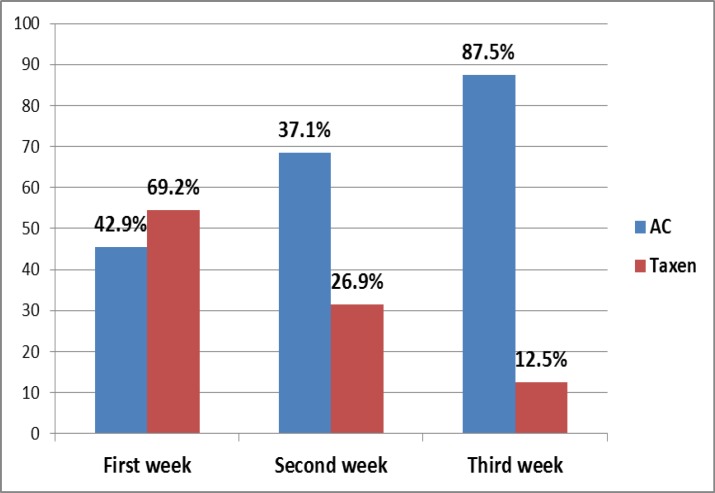
Distribution of OM patients by chemotherapy protocols and period of OM appearance

Figure 5 shows the appearance of OM signs with Taxane in the first week of chemotherapy cycle is higher (69.2%) than those with AC protocol (42.9%). Statistically, there is a significant association between type of protocol and time of appearance of OM signs (p=0.05). Score 2 is the most prominent in the first and second week of the cycle after treatment (51.5% and 60%, respectively) while the 3rd score occurrence decreases with increasing time after treatment till it is not occur in the 3rd week.

**Figure 6:**
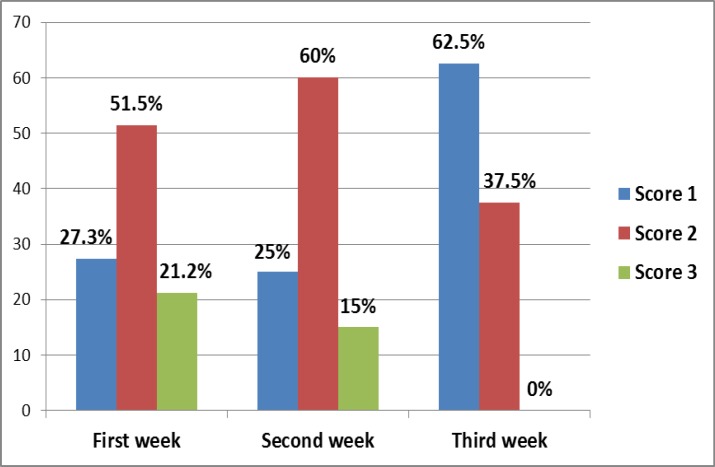
Distribution of OM patients by OM score and period of its appearance

This association was not statistically significant (Figure 6).

## Discussion

 Mucositis has been of interest to scientists for more than 20 years. Unfortunately, this has not resulted in development of standard procedures for prevention and management.^7^

A comprehensive understanding of Oral Mucositis pathogenesis, together with a clear definition of risk factors for development and severity of the lesion, remain under investigation.^8^ In present study, 81.3% of patients with breast cancer who received chemotherapy (Adriamycine, Cychlophosphamid and Taxane) in onco-radiotherapy department in Al-Bashir hospital were suffering from OM. Köstler WJ and his colleagues in their study showed that incidence of OM may reach up to 40% of patients treated with conventional chemotherapy.^9^ But in Dodd and his colleagues study were shown that OM developed in (25.1%) of study sample.^10^

Otmani and his colleagues study in 2008 found OM in 65.4% of study population but it was conducted in Pediatrics age group (mean age 6.82 ± 4.08 years).^11^ The closest results found in Chen HM study in 2008 which 75.4% of participants (N=57) had experienced at least one episode of OM since their first chemotherapy.^12^

The relative higher percentage in our study can relate to (single sign) diagnosis system that used to detect cases of OM. Also, it may be related to difference in scoring system that used in comparison with other studies.

(16%) and (30%) of our patients were suffering from leukopenia and neutropenia, respectively. There were no significant associations between the total WBC count and neutrophil count with OM occurrence in our study. This result did not correlate with results that found by Suresh AV and his colleagues in 2010. They found a positive correlation between the markers of local immunity (total WBC counts, comorbid conditions, tobacco use and nutritional status as reflected by the albumin levels). This difference may be due to the spectrum of immunity markers that used, in which we used just 2 markers in compare with 4 that were used in Suresh et al. study in 2010.^13^

There are conflicting data related to the effects of age and the development of chemotherapy-induced Mucositis.^14^ In general, younger patients appear to have an increased risk of chemotherapy-induced Mucositis. This observation may be explained by the more rapid epithelial mitotic rate or the presence of more epidermal growth factor receptors in the epithelium of younger patients. Alternatively, the physiologic decline in renal function associated with aging may result in older patients being at higher risk of chemotherapy-induced Mucositis.^15^ In present study, our results go with the second opinion. Table 2 was shown that the higher occurrence of OM found in age category of more than 45 year but it was not significant association which may relate to renal function test. However, there is no significant difference between means of S. creatinine which reflect the situation of renal function of study sample. In conjunction with patient-related factors, factors that are treatment-related include specific chemotherapeutic drug, dose, schedule and use of radiation therapy.^16^

All of these were affected the subsequent development (severity and duration) of Mucositis. Protracted infusions of anti metabolites concomitant as well as using of radiation, result in more severe Mucositis.^14^ In our study, 81.4% of patients who were on Adriamycin and 89.3% of those who were on Taxane suffered from OM. The association between type of chemotherapy protocol and occurrence of OM was significant (p=0.009). On the other hand, Sonis et al. study showed that with conventional chemotherapy including Anthracycline-based regimens, Taxane-based regimes and platinum-based regimens, severe Oral Mucositis occurs in 1 to 10 percent of patients but this can go as high as 66 percent when these agents are combined with 5 fluorouracil (5-FU). 5-FU alone typically causes severe Oral Mucositis in over 15 percent of patients.^17^

In Raber-Durlacheret et al. study in 2000 found that 16 patients (34%) only suffering from slight oral mucosal changes were recorded (maximum WHO score 1), while 25 patients (53.1 %) experienced mild to moderate Mucositis (maximum WHO score 2) and in 6 patients (12.9%) Mucositis was moderate to severe (maximum WHO score 3). No grade 4 Mucositis developed.^18^ This results were compatible with our results in which we registered that 31.3%, 52.4% and 16.3 of patients were experienced score I, II and III, respectively. Moreover, no grade 4 was registered. Oral Mucositis began 5-10 days following the initiation of chemotherapy and persists for 7-14 days. Therefore, the whole process persists for 2-3 weeks in more than 90% of patients.^15^ This is similar to what we found in our study in which more than half of the cases started OM in the first week (2–7 days) and 41.3% of cases appeared in the second cycle of chemotherapy.

In literature review, many studies have shown a correlation between the increase of OM incidence (especially moderate to severe OM) after chemo and radiotherapy administration with low BMI.^18^^-^^20^ However, our study shows the opposite results. The incidence of OM is raised with increasing of BMI but the association between BMI and OM was not significant.

## CONCLUSION

 Oral Mucositis is one the most common problem that may have impact on QOL of patients who receive chemotherapy. Defining the most important factors (patient-related and chemotherapy-related) that associated with occurrence of (OM) were very useful to improve patients' performance status during chemotherapy cycles.

Nurses play a key role in determination of risk factors that considered significant predictors of the severity of OM and teaching patients to detect sign of OM and overcome its effects. Using of Taxane including protocols can be considered as the most important significant risk factor which increase incidence of OM in this study. Therefore, future longitudinal studies using probability sampling methods are highly recommended to generalize the study's results.
